# Personal

**Published:** 1900-06

**Authors:** 


### PERSONAL.

Dr. Roswell Park, of Buffalo, was elected president of the American Surgical Association at its recent annual meeting held at Washington, D.C. Dr. Park, we are pleased to learn, is convalescing from his recent illness and is sojourning at Atlantic City, for a little time. Just as these pages are printing we learn that Dr. Park has been appointed Medical Director of the Pan-American Exposition.

Dr. Eli H. Long, of Buffalo, was elected one of the vice-presidents of the American Therapeutic Society, which was lately organised at Washington, D.C. The next meeting is called at Washington on May 7, 1901.

Dr. Myrtle A. Hoag announces her removal from 25 Wadsworth Street to 397 Dearborn Street, Buffalo. Office hours, 2.30 to 3.30 and 7 to 8 p.m.

Dr. J. Glenn Ernest, of Buffalo, has removed from 295 Goodyear Avenue to 115 Northampton Street. Hours: 8 to 9 a. m.; 1 to 3 and 7 p.m.

Dr. Grosvenor R. Trowbridge, of Buffalo, has removed his offices from Ellicott Square to The Wellesley, cor. of Franklin and Edward Streets.

Dr. Henry Nichell, of Buffalo, has removed from 80 Sycamore Street to No. 27 Day's Park.

